# Natural Fermentation Quality and Bacterial Community of 12 *Pennisetum sinese* Varieties in Southern China

**DOI:** 10.3389/fmicb.2021.627820

**Published:** 2021-04-29

**Authors:** Xuejuan Zi, Mao Li, Daogeng Yu, Jun Tang, Hanlin Zhou, Yeyuan Chen

**Affiliations:** ^1^Key Laboratory of Ministry of Education for Genetics and Germplasm Innovation of Tropical Special Trees and Ornamental Plants, Key Laboratory of Germplasm Resources of Tropical Special Ornamental Plants of Hainan Province, College of Forestry, College of Tropical Crops, Hainan University, Danzhou, China; ^2^Tropical Crops Genetic Resources Institute, Chinese Academy of Tropical Agricultural Sciences, Danzhou, China

**Keywords:** *Pennisetum sinese*, varieties, silage, fermentation quality, bacterial community, environmental factor

## Abstract

This study investigated the fermentation quality of 12 varieties of *Pennisetum sinese* grown in different regions of Southern China. Following the production of silage from the natural fermentation of *P. sinese*, the interplay between the chemical composition, fermentation characteristics, environmental factors, and microbiome was examined to understand the influence of these factors on the fermentation quality of silage. The silage quality produced by most of the *P. sinese* was low; the pH value of the silage was high (4.26–4.86), whilst the lactic acid content was low (10.7–24.1 g/kg DM), with V-scores between 57.9 and 78.3. The bacterial alpha diversities of the 12 *P. sinese* silages were distinct. There was a predominance of undesirable bacteria (*Pseudomonas*, *Massilia*, and *Raoultella)*, which likely caused the poor fermentation quality. The chemical composition and fermentation characteristics of the silage were closely correlated with the composition of the bacterial community. Furthermore, environmental factors (precipitation, temperature, humidity, location) were found to significantly influence the microbiome of the silage. The results confirmed that silage produced from the natural fermentation of 12 different *P. sinese* varieties had significant variation in their bacterial communities. The difference in environmental factors, due to the *P. sinese* being grown in various locations across south china, greatly affected the bacterial community found in the silage and thus the fermentation quality. The specific cultivar used for the silage and the environment in which the cultivar is grown must therefore be considered before the initiation of production of silage in order to ensure a higher quality product.

## Introduction

*Pennisetum sinese* is a member of the grass family. It is adaptable and has a high biomass production rate; this has led to its widespread use in tropical/subtropical regions across the world (Zhao et al., 2019). *P. sinese* is primarily used for the production of feedstock for biofuels and animal feed; this is due to its high lignocellulosic biomass and excellent palatability (Li et al., 2014, 2019a,2019b, 2020; Lu et al., 2014; Wang et al., 2016; Shah et al., 2018). The demand for *P. sinese* is large, but production is affected by seasonal harvest. The summer/rainy seasons see vigorous *P. sinese* growth, with a contrasting lack of growth during winter and times of drought; this leads to a shortage in its availability (Li et al., 2014). In order to combat this and meet the year-round demand, *P. sinese* needs proper pretreatment and conservation.

*Pennisetum sinese* has a high moisture content, making the plant susceptible to mildew and rot; this causes biomass decomposition and inefficient biotransformation. Those using *P. sinese* in tropical regions face considerable challenges regarding preservation and ineffective biotransformation, due to the hot and humid environment. Ensiling is a method that can conserve green plants by utilizing anaerobic fermentation. Ensiling can guarantee the quality of the feed for long term storage and has also been shown to be a promising pretreatment method in biofuel production (Wilkinson et al., 2003). Previous studies have focused primarily on the effect of additives to *P. sinese* silages, but little is currently understood regarding the effect of additives on the microbiome of the resultant silage (Li et al., 2014, 2018a,2018b,2019a, 2020; Li L. et al., 2017; Wu et al., 2020). Currently, only a limited number of studies have examined the effects of additives on the fermentation quality and microbial diversity of *P. sinese* silage (Dong et al., 2020; Shah et al., 2020). It is of note that these studies have focused on specific *P. sinese* cultivars.

As previously mentioned, uncontrollable environmental factors challenge the production of silage in tropical areas (Bernardes et al., 2018). The significance of errant weather conditions has previously been examined in silage produced from the fermentation of Napier and Italian Ryegrass (Guo et al., 2014, 2015). In addition, found that environmental factors influenced the bacterial diversity of corn silage and thus altered the fermentation quality (Guan et al., 2018). Moreover, it has been reported that temperature can influence the bacterial diversity and fermentation quality of silage produced from *Moringa oleifera* (Wang et al., 2019a).

*Pennisetum sinese* is grown across multiple regions of southern china that differ greatly in climate. Therefore, the influence of environmental factors on silage quality and microbial diversity should be examined and any effects should be investigated in the multiple *P. sinese* cultivars that are used across the regions.

This study aimed to examine the differing bacterial communities and the fermentation of silages produced from 12 different *P. sinese* varieties. The *P. sinese* studied were grown across multiple regions in southern China; this allowed the experimental parameters to include influence of environmental factors (precipitation, temperature, humidity, location) on the silage microbiome across a broad spectrum of varieties of *P. sinese*.

## Materials and Methods

### Silage Preparation

Twelve *P. sinese* varieties were grown at six different bases across southern China; the location and environmental factors related to these bases are shown in Supplementary Table 1. The *P. sinese* cultivars used in this study were planted on the 5th of April 2019 and then harvested on June 26th 2019. The *P. sinese* material was immediately chopped into small pieces (∼2 cm). Briefly, 200 g of each grass sample was vacuum-packed into plastic bags. A total of 36 bags (twelve varieties in triplicate) were prepared and incubated at 25–30°C for 30 days. The organic acid content and bacterial community of the silage was then determined.

### Chemical Composition and Fermentation Analysis

Specimens were dried at 65°C for 2 days and passed through a 1.0 mm sieve before the chemical assay. The contents of dry matter (DM), crude protein (CP), organic matter (OM), and ether extracts (EE) were examined according to previously published work (AOAC, 1990). Moreover, the contents of neutral detergent fiber (NDF) and acid detergent fiber (ADF) were assessed using a previously established method (Van Soest et al., 1991). Heat-stable amylase and sodium sulfite were adopted in the determination of NDF. Water-soluble carbohydrate (WSC) was determined according to a previously described method (Murphy, 1958).

The fermentation quality of the silage was determined using distilled water extracts. 50 g of wet silage was blended with 200 mL distilled water, before 24 h of incubation (4°C) and filtration. The pH, lactic acid, acetic acid, propionic acid, butyric acid, and ammonia-N concentrations were measured as previously established (Li et al., 2019b). To evaluate the silage quality the V-score was calculated using the formula: ammonia-N/Total N and organic acid (Masaki, 2001).

### Microbial Community Analysis

The above-mentioned extracts were used for molecular analysis of the silage microbiome. Briefly, 20 mL filtrate was centrifuged at 12,000 *g*/min for 5 min, and the sediment was collected from the bottom. Microbial DNA was isolated from silage specimens using the E.Z.N.A.^®^, soil DNA Kit (Omega Bio-Tek, Norcross, GA, United States), according to the manufacturer’s instructions. The concentration and purity of the extracted DNA was assessed using a NanoDrop 2000 UV-Vis spectrophotometer (Thermo Fisher Scientific, Wilmington, DE, United States) and the integrity of the DNA was assessed by electrophoresis on a 1% agarose gel. A thermocycler PCR system (GeneAmp 9700, ABI, United States) and primers 338F (5′-ACTCCTACGGGAGGCAGCAG-3′) and 806R (5′-GGACTACHVGGGTWTCTAAT-3′) were used to amplify the V3–V4 hypervariable regions of the bacterial 16S rRNA gene. After the PCR products were purified and quantified, next-generation sequencing was carried out using an Illumina MiSeq 2500 platform (Illumina, Inc., San Diego, CA, United States). Paired-end reads of 250 bp were generated.

Tag assembly was carried out using filtered reads according to the following principles: the overlap between paired-end reads should be more than 10-bp and have less than a 2% mismatch. The unique tags were obtained by removal of redundant tags using MOTHUR software (Schloss et al., 2009). Abundancy was determined using the resultant unique tags and high-quality reads were grouped into operational taxonomic units (OTUs) defined as having 97% similarity. Diversity metrics were determined using the core-diversity plug-in within QIIME2^1^ (Callahan et al., 2016). The microbial diversity within an individual sample was assessed using the following alpha diversity indices: the Chao 1 richness estimator, Shannon diversity index, and Faith’s phylogenetics diversity index. Beta diversity was analyzed to assess the structural variation of microbiota across *P. sinese* specimens. Subsequently, principal component analysis (PCA) was undertaken (Vázquez-Baeza et al., 2013). Appropriate methods were employed to identify the abundances of different bacterial strains amongst samples and groups as described previously (Segata et al., 2011). Unless specified above, parameters used in the genetic analysis were set as default. The heat map function of the R software^2^ and genus information for the *P. sinese* silage were used to generate a heat map. The data were analyzed using the free online BMKCloud Platform^3^. The sequencing data was submitted to the National Center for Biotechnology Information Sequence Read Archive database under the BioProject accession number PRJNA624770.

### Statistics

The impact of variety was investigated using one-way analysis of variance (SAS 9.3 software, SAS Institute Inc., Cary, NC, United States). Significant differences were compared using Duncan’s multiple range tests, with *P* < 0.05 being regarded as statistically significant.

## Results

### The Chemical Composition of Fresh *P. sinese*

The Chemical compositions of different fresh *P. sinese* samples are shown in Table 1. In this study, the *P. sinese* DM content ranged from 113.6 to 225.2 g/kg; the MZC and MOTT samples had the highest and lowest DM contents, respectively (*P* < 0.05). The CP contents ranged from 47.4 to 73.0 g/kg, with the MZC and JING samples (*P* < 0.05), having the highest and lowest CP values, respectively. The OM contents of all *P. sinese* samples were similar, above 900 g/kg, with no significant difference. The NDF content of all the samples ranged from 522.0 to 652.8 g/kg; the highest and lowest levels were found in the PURP and XC samples, respectively (*P* < 0.05). The ADF content of the *P. sinese* samples ranged from 230.6 to 391.8 g/kg; the highest and lowest levels were found in the MOTT and XC samples, respectively (*P* < 0.05). The WSC content of the *P. sinese* samples ranged from 53.8 to 93.4 g/kg, with the highest levels in the MIN and DY samples, respectively (*P* < 0.05). Overall, with the exception of the OM content, the chemical composition of the *P. sinese* samples varied greatly.

**TABLE 1:** Chemical composition of 12 *P. sinese* varieties (g/kg DM^–1^).

**Variety**	**DM**	**CP**	**OM**	**NDF**	**ADF**	**WSC**
MIN	209.1^ab^	62.1^b^	949.3^a^	647.5^a^	377.0^b^	93.4^a^
TW	124.3^f^	54.6^*cd*^	936.9^a^	537.2^d^	342.3^bc^	96.3^a^
MOTT	113.6^g^	58.2^bc^	941.2^a^	608.5^b^	391.8^a^	68.8^c^
KG	186.8^c^	55.9^c^	919.8^a^	605.1^b^	358.3^b^	80.6^b^
DY	181.5^c^	58.3^bc^	930.6^a^	584.2^bc^	333.7^c^	53.8^e^
HONG	129.8^f^	65.9^b^	917.3^a^	575.4^c^	356.9^b^	71.0^c^
JING	183.2^c^	47.4^f^	902.4^a^	569.2^c^	347.5^bc^	73.6^c^
AX	128.7^f^	58.4^bc^	936.7^a^	525.7^e^	230.6^d^	57.2^d^
ZJ	201.7^b^	53.6^d^	913.6^a^	599.7^b^	357.0^b^	62.4^d^
MZC	225.2^a^	73.0^a^	930.6^a^	588.6^bc^	366.0^b^	90.3^a^
PURP	145.0^e^	51.7^e^	946.1^a^	652.8^a^	360.8^b^	60.1^d^
XC	163.4^d^	56.6^c^	919.1^a^	522.0^e^	366.9^b^	63.4^d^
SEM	10.76	1.94	4.13	12.26	11.65	4.20
*P*-value	<0.01	<0.01	<0.01	<0.01	<0.01	<0.01

*MIN, *Pennisetum purpureum* Schum. cv. Gui Min Yin; TW, *Pennisetum purpureum* Schumab ev. Taiwan; MOTT, *Pennisetum purpureum* cv. Mott; KG, *Pennisetum purpureum* × *P*. *glaucum* cv. Reyan No. 4; DY, *Pennisetum purpureum* K.; HONG, *Pennisetum purpureum* Schumab cv.; JING, *Pennisetum purpureum* K. Jingmu No. 1; AX, *Pennisetum purpureum* K.; ZJ, *Pennisetum americanum* × *Pennisetum purpureum*; MZC, *Pennisetum glaucum*; PURP, *Pennisetum purpureum* cv. Purple; XC, *Pennisetum purpureum* Schumach. DM, dry matter, CP, crude protein, OM, organic matter; NDF, neutral detergent fiber; ADF, acid detergent fiber; WSC, water-soluble carbohydrate; SEM, standard error of means. Means within the same column with different letters are significantly different (*P* < 0.05).*

### The Fermentation Property of *P. sinese* Silage

The fermentation properties of silage produced by different *P. sinese* varieties are shown in Table 2. There were significant differences in the pH levels, as well as the lactic acid, acetic acid, propionic acid, butyric acid, and ammonia-N content depending on the variety off *P. sinese* used to produce the silage (*P* < 0.05). The pH of the silages ranged from 4.36 to 4.86, with the silage produced by MIN and MZC *P. sinese* varieties having the highest and lowest pH levels, respectively (*P* < 0.05). The lactic acid content of all *P. sinese* silages ranged from 10.7 to 24.1 g/kg, with XC having the highest lactic acid content and AX the lowest (*P* < 0.05). The acetic acid content of the different silages ranged from 3.0 to 16.6 g/kg, with the silages produced by the MOTT and AX *P. sinese* samples having the highest and lowest levels, respectively (*P* < 0.05). The propionic acid content of all *P. sinese* silage samples ranged from 1.1 to 17.3 g/kg; the highest propionic acid content was found in the XC (*P* < 0.05), whilst the lowest propionic acid content was found in the MZC sample (*P* < 0.05). Meanwhile, the butyric acid content of all *P. sinese* silage samples ranged from 1.2 to 4.6 g/kg. The silage from the MIN sample had the highest butyric acid content with the PURP sample silage having the lowest (*P* < 0.05). Finally, the ammonia-N content of the *P. sinese* silages ranged from 44.5 to 67.2 g/kg, the highest ammonia-N content was found in the JING silage sample (*P* < 0.05), whilst the lowest in the ZJ sample (*P* < 0.05). The V-Scores of all the *P. sinese* silages ranged from 57.9 to 78.3, the highest V-Score was found in the AX sample silage with the lowest in the MIN sample (*P* < 0.05). All the naturally fermented *P. sinese* silages in this study had a low V-score, which suggests that the silage quality was unsatisfactory. In addition, the results showed large variability in the quality of fermentation and V-scores across the different *P. sinese* silage samples.

**TABLE 2:** Fermentation characteristics of the silage from 12 varieties of *P. sinese* (g/kg DM^–1^).

**Variety**	**pH**	**Lactic acid**	**Acetic acid**	**Propionic acid**	**Butyric acid**	**Ammonia -N**	**V-Score**
MIN	4.86^a^	16.6^c^	3.8^g^	11.1^*cd*^	3.6^a^	66.7^a^	57.9^d^
TW	4.57^b^	15.4c	7.6e	12.6c	2.4^bc^	54.2^bc^	70.0^b^
MOTT	4.75^ab^	21.6^b^	16.6^a^	15.7^b^	2.3^bc^	60.8^b^	69.4^b^
KG	4.39^*cd*^	13.7^d^	5.7^f^	9.5^d^	2.7^b^	45.8^d^	68.4^b^
DY	4.68^ab^	12.4^e^	5.0^f^	6.5^e^	1.9^d^	49.2^c^	77.5^a^
HONG	4.54^bc^	12.2^e^	9.4^d^	1.4^f^	3.9^a^	53.5^bc^	61.3^*cd*^
JING	4.81^a^	16.1^c^	13.9^b^	12.4^c^	3.1^ab^	67.2^a^	61.8^*cd*^
AX	4.62^b^	10.7^f^	3.0^g^	2.0^f^	2.2^bc^	58.8^b^	78.3^a^
ZJ	4.46^c^	12.4^e^	8.1^e^	6.0^e^	2.6^b^	44.5^d^	69.9^b^
MZC	4.36^d^	20.4^b^	10.6^*cd*^	1.1^f^	2.6^b^	48.3^c^	71.7^b^
PURP	4.63^b^	12.8^e^	6.3^f^	13.0^c^	2.2^bc^	51.0^c^	72.2^b^
XC	4.50^bc^	24.1^a^	11.5^c^	17.3^a^	3.4^a^	49.8^c^	62.8^*cd*^
SEM	0.05	1.23	1.19	1.61	0.18	2.21	1.84
*P*-value	<0.05	<0.01	<0.01	<0.01	<0.01	<0.01	<0.01

*MIN, *Pennisetum purpureum* Schum. cv. Gui Min Yin; TW, *Pennisetum purpureum* Schumab ev. Taiwan; MOTT, *Pennisetum purpureum* cv. Mott; KG, *Pennisetum purpureum* × *P*. *glaucum* cv. Reyan No. 4; DY, *Pennisetum purpureum* K.; HONG, *Pennisetum purpureum* Schumab cv.; JING, *Pennisetum purpureum* K. Jingmu No. 1; AX, *Pennisetum purpureum* K.; ZJ, *Pennisetum americanum* × *Pennisetum Purpureum*; MZC, *Pennisetum glaucum*; PURP, *Pennisetum purpureum* cv. Purple; XC, *Pennisetum purpureum* Schumach; SEM, standard error of means. Means within the same column with different letters are significantly different (*P* < 0.05).*

### Bacterial Community of *P. sinese* Silage

A total of 2,677,149 raw reads were generated, with an average of 71,884 raw tags per *P. sinese* sample. After data processing, there was an average of 71,423 clean tags and 60,717 effective tags obtained from the silage samples.

**FIGURE 1:**
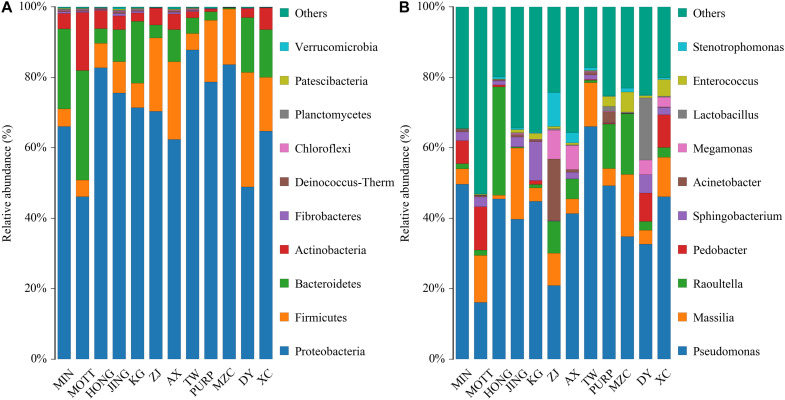
Relative abundance of bacterial composition in *P. sinese* silages at phylum **(A)** and genus **(B)** level.

Table 3 shows the alpha diversity of the silage bacterial communities. The OTU, Shannon, Simpson, Ace, and Chao 1 indices of microbial diversity were affected by the variety of samples (Table 3). The OTUs, Shannon, Simpson, Ace, and Chao 1 values ranged from 383 to 500, 1.39 to 3.54, 0.08 to 0.45, 367.57 to 476.25, 368.29 to 490.58, respectively. In conclusion, the variety of *P. sinese* greatly affected the microbiome of the resultant silage. Figure 1A describes the microbial community at the phylum level. *Proteobacteria, Firmicutes, Bacteroidetes*, and Actinobacteria were dominant in all *P. sinese* samples. To further investigate the effects of *P. sinese* variety on the resultant microbial community following ensiling, the bacterial structures of *P. sinese* silages at the genus level were examined (Figure 1B). The 10 genera with the highest relative abundance were: *Pseudomonas*, *Massilia*, *Raoultella*, *Pedobacter*, *Sphingobacterium*, *Acinetobacter*, *Megamonas*, *Lactobacillus*, *Enterococcus*, and *Stenotrophomonas*. The present study shows that *Pseudomonas* is one of the most abundant genera of bacteria present in the silages produced by differing *P. sinese* varieties. It is proposed that *Pseudomonas* contributed to the poor *P. sinese* silage quality.

**TABLE 3:** The bacterial alpha diversity of silage from 12 *P. sinese* varieties.

**Variety**	**OTUs**	**Shannon**	**Simpson**	**ACE**	**Chao 1**	**Coverage**
MIN	489	2.97	0.25	461.46	468.44	0.99
TW	491	2.51	0.22	463.36	466.13	0.99
MOTT	486	3.54	0.08	469.19	477.53	0.99
KG	489	2.59	0.30	464.63	474.68	0.99
DY	419	2.59	0.16	460.27	382.71	0.99
HONG	500	1.96	0.45	469.16	473.36	0.99
JING	497	3.42	0.16	476.25	481.40	0.99
AX	495	3.23	0.20	475.82	490.58	0.99
ZJ	469	2.64	0.17	434.45	423.30	0.99
MZC	383	1.39	0.44	367.57	368.29	0.99
PURP	421	2.27	0.23	430.28	395.26	0.99
XC	406	2.53	0.17	368.59	372.94	0.99

*MIN, *Pennisetum purpureum* Schum. cv. Gui Min Yin; TW, *Pennisetum purpureum* Schumab ev. Taiwan; MOTT, *Pennisetum purpureum* cv. Mott; KG, *Pennisetum purpureum* × *P*. *glaucum* cv. Reyan No. 4; DY, *Pennisetum purpureum* K.; HONG, *Pennisetum purpureum* Schumab cv.; JING, *Pennisetum purpureum* K. Jingmu No. 1; AX, *Pennisetum purpureum* K.; ZJ, *Pennisetum americanum* × *Pennisetum Purpureum*; MZC, *Pennisetum glaucum*; PURP, *Pennisetum purpureum* cv. Purple; XC, *Pennisetum purpureum* Schumach.*

**FIGURE 2:**
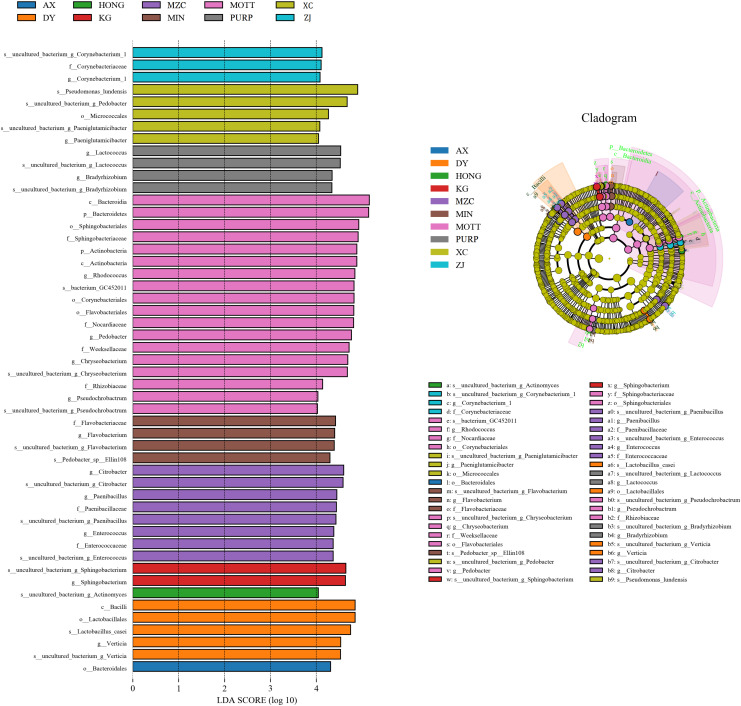
Comparison of microbial variations in *P. sinese* silage using the LEfSe online tool.

The linear discriminant analysis (LDA) effect size (LEfSe) method was used to assess the differences in the microbiome content between the silages produced by the different *P. sinese* varieties, as well as to explore the specific bacterial community of each silage variety (LDA score >4.0). Figure 2 shows that the variety of *P. sinese* used to produce the silage had a dramatic impact on the resultant microbial community. *Bacteroidales* was the most abundant order in the silage of the AX *P. sinese* variety, *Verticia* was the most abundant genus and *Lactobacillus casei* was the most abundant species in the DY silage. *Actinomyces* was the most abundant species in the HONG silage, *Sphingobacterium* was the most abundant genus in the KG silage whilst *Enterococcus*, *Paenibacillus*, and *Citrobacter* were the most abundant genera in the MZC silage. *Flavobacterium* was the most abundant genus and *Pedobacter Ellin108* the most abundant species in the MIN silage. *Corynebacteriales* and *Flavobacteriales* were the most abundant orders, *Sphingobacteriaceae*, *Nocardiopsaceae*, *Weeksellaceae*, and *Rhizobiaceae* the most abundant families and *Enterococcus*, *Pedobacter*, *Chryseobacterium*, and *Pseudochrobactrum* were the most abundant genera in the MOTT silage. Conversely, *Lactococcus*, and *Bradyrhizobium* were the most abundant genera in the PURP silage, whilst in the XC silage *Micrococcales* was the most abundant order, *Paeniglutamicibacter* the most abundant genus and *Pseudomonas lundensis* was the most abundant species. Finally, *Corynebacterium_1* was the most abundant genus in ZJ silage. It is proposed that these microorganisms could be used as biomarkers for the silages produced by the different varieties of *P. sinese*.

### Association Analysis Between Bacterial Communities and the Chemical Composition of Raw *Pennisetum sinese* Before Ensiling

**FIGURE 3:**
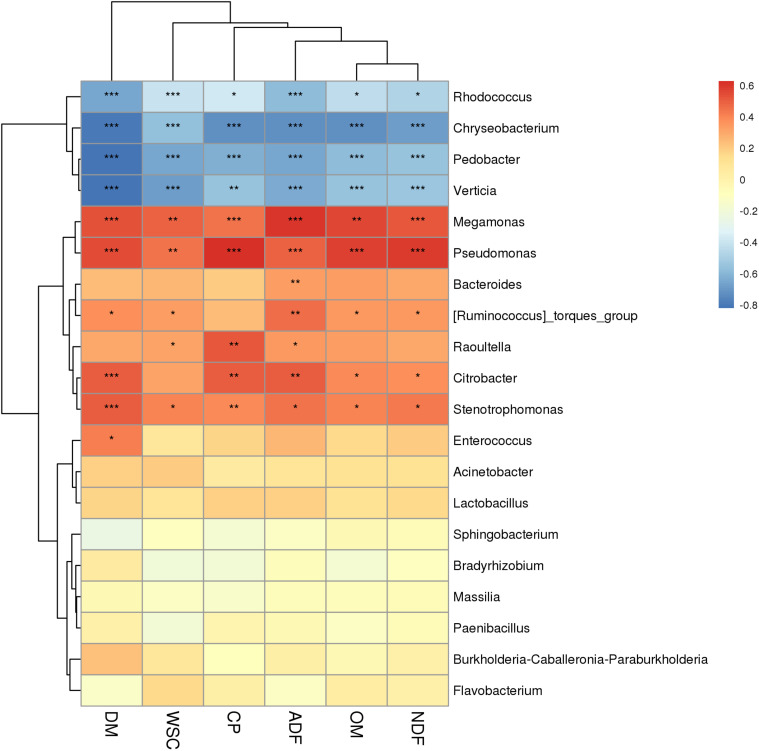
Correlation analysis of the bacterial community with chemical composition of *P. sinese* silage at genus level. The blue indicates a negative correlation, red indicates a positive correlation. “*,” “**,” and “***” represent *P* < 0.05, *P* < 0.01, and *P* < 0.001, respectively.

The correlation between chemical composition (DM, OM, CP, NDF, ADF, and WSC) and the microbiome of *P. sinese* silages was also assessed. At the genus level, a Spearman correlation heat map was created for the microbial communities from the silages (Figure 3). *Megamonas*, *Pseudomonas*, *Bacteroides*, *Raoultella*, *Citrobacter*, *Stenotrophomonas*, and *Enterococcus* abundance positively correlated with the DM, OM, CP, NDF, ADF, and WSC content (*P* < 0.05). In addition, *Rhodococcus*, *Chryseobacterium*, *Pedobacter*, and *Verticia* abundance was negatively correlated with these chemical compositions of raw *P. sinese* (*P* < 0.05). This present study showed significant correlation between the species of bacteria and the chemical composition of the raw *P. sinese* used to produce the silage. The results of this study further compound the significant relationship between the chemical composition of the raw material and the resultant silage.

### Association Analysis Between Bacterial Communities and Fermentation Characteristics

**FIGURE 4:**
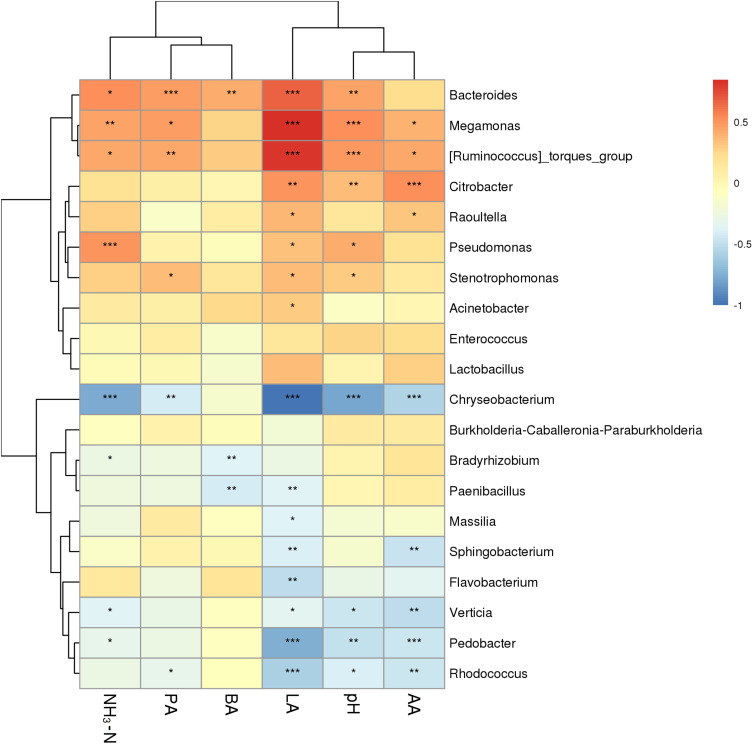
Correlation analysis of *P. sinese* silage bacterial communities and fermentation characteristics at genus level. The blue indicates a negative correlation, red indicates a positive correlation. “*,” “**,” and “***” represent *P* < 0.05, *P* < 0.01, and *P* < 0.001, respectively.

The correlation between fermentation characteristics and the microbiome of *P. sinese* silages was assessed (Figure 4). Silage pH was positively correlated with the abundance of *Bacteroides*, *Megamonas*, and *Citrobacter* (*P* < 0.01) in the silage microbiome, while it was negatively correlated with the abundance of *Chryseobacterium* and *Pedobacter* (*P* < 0.01). The silage lactic acid content was positively correlated with the abundance of *Bacteroides*, *Megamonas*, and *Citrobacter* (*P* < 0.01), while it was negatively correlated with the abundance of *Chryseobacterium*, *Paenibacillus*, *Sphingobacterium*, *Flavobacterium*, *Pedobacter*, and *Rhodococcus* (*P* < 0.01). The acetic acid content of the silage was positively correlated with the abundance of *Citrobacter* (*P* < 0.01), while it was negatively correlated with the abundance of *Chryseobacterium*, *Sphingobacterium*, *Verticia*, *Pedobacter*, and *Rhodococcus* (*P* < 0.01). The propionic acid content of the silage was positively correlated with the abundance of *Bacteroides* (*P* < 0.01), while it was negatively correlated with the abundance of *Chryseobacterium* (*P* < 0.01). Finally, the butyric acid content of the silage was positively correlated with the abundance of *Bacteroides* (*P* < 0.01), while it was negatively correlated with the abundance of *Bradyrhizobium* and *Paenibacillus* (*P* < 0.01).

### Association Analysis Between Bacterial Communities and Environmental Factors

**FIGURE 5:**
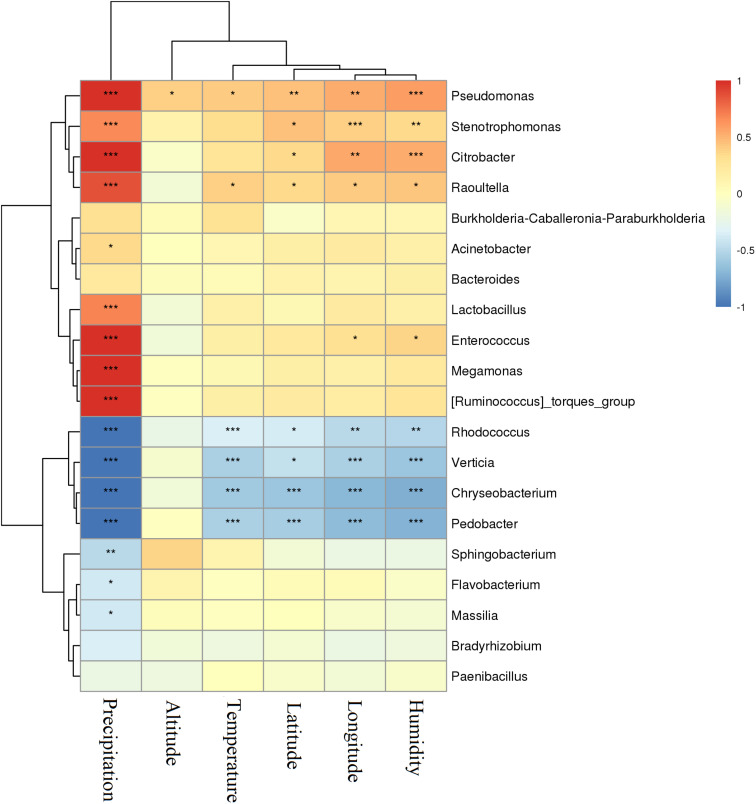
Correlation of *P. sinese* silage bacterial communities and environmental factors (longitude, latitude, temperature, humidity, and precipitation) at genus level. The blue indicates a negative correlation, red indicates a positive correlation. “*” and “**” represent *P* < 0.05 and *P* < 0.01, respectively.

The relationship between environmental factors the varying *P. sinese* samples were exposed to Supplementary Table 1 and the silage they produced was assessed (Figure 5). The level of precipitation the *P. sinese* sample was exposed to was positively correlated with the abundance of *Pseudomonas, Stenotrophomonas, Citrobacter, Raoultella, Lactobacillus, Enterococcus*, and *Megamonas* (*P* < 0.001), whilst it was negatively correlated with the abundance of *Rhodococcus, Verticia, Chryseobacterium*, *Pedobacter*, and *Sphingobacterium* (*P* < 0.01). The environmental temperature was positively correlated with the abundance of *Pseudomonas* and *Raoultella* (*P* < 0.05), whilst it was negatively correlated with the abundance of *Rhodococcus, Verticia, Chryseobacterium*, and *Pedobacter* (*P* < 0.01). Meanwhile, environmental humidity was positively correlated with the abundance of *Pseudomonas, Stenotrophomonas*, and *Citrobacter* (*P* < 0.01), whilst it was negatively correlated with the abundance of *Rhodococcus, Verticia* and *Chryseobacterium*, *Pedobacter* (*P* < 0.01). The longitude and latitude of the base in which the *P. sinese* sample was grown was positively correlated with the abundance of *Pseudomonas, Stenotrophomonas, Citrobacter*, and *Raoultella* (*P* < 0.05), whilst it was negatively correlated with the abundance of *Rhodococcus, Verticia, Chryseobacterium*, and *Pedobacter* (*P* < 0.05). The altitude at which the *P. sinese* sample was grown was positively correlated with the abundance of *Pseudomonas* but no other bacteria, suggesting that altitude played a only a minor role in determining the microbiome content of the silage (*P* < 0.05).

## Discussion

### Characteristics of *P. sinese* Before Ensiling

The chemical composition of raw materials has an important influence on the silage quality. In this study, the chemical composition of *P. sinese* varieties are similar to a previous study in which king grass was examined (Li et al., 2019b). In contrast, both Mugabe et al. (2019) and Li et al. (2020) reported higher CP, NDF, and ADF content in *P. sinese* varieties compared to the results described. Furthermore, Desta et al. (2016) reported a higher DM, NDF, and ADF content, but lower WSC content, in Napier grass. Shah et al. (2017) also found a higher NDF content, whilst they also found a lower CP and WSC content in king grass compared to the results presented herein. These results imply that there are a large differences in the chemical compositions of different varieties of *P. sinese*; this could influence the silage quality. In particular, low levels of WSC are thought to contribute to the poor fermentation quality of *P. sinese* silage without additives (Wang et al., 2019a).

### The Fermentation Properties of *P. sinese* Silage

Silage pH is a very important evaluation parameter to consider when assessing fermentation efficacy; well fermented silage should have a pH of 4.2 or lower (Edwards and McDonald, 1978). The results presented in this study show that all silages had pH of higher than 4.2, suggesting a lower quality of fermentation. Previous studies have reported a range of silage pH’s with some corroborating what was found in this study and other papers showing *P. sinese* silage with a lower pH (Li et al., 2014, 2019a, 2020; Desta et al., 2016; Mugabe et al., 2019). The pH variability may be due to different *P. sinese* varieties being used in this study compared to others, or differences in the silage microbiome. This study revealed relatively low lactic and propionic acid content compared to previous studies. Meanwhile, the acetic acid, butyric acid, and ammonia-N content were similar to, or lower than, the levels reported in previous papers looking at silage produced from *P. sinese* grass (Li et al., 2014, 2019a,2019b, 2020; Desta et al., 2016; Shah et al., 2017; Mugabe et al., 2019). The differing chemical compositions of the raw *P. sinese* samples may have contributed to the substantial variation in silage fermentation characteristics; Guan et al. (2018) reported this phenomenon previously, albeit in corn silage. Desta et al. (2016) reported the V-Score of Napier grass silage without additive as 63.1, this falls within the range elucidated in this study. All the naturally fermented *P. sinese* silages in this study had a low V-score, which suggests that the silage quality was unsatisfactory. The poor fermentation quality may be due to the low levels of WSC. WSC is an important substrate for lactic acid bacteria (LAB); these are the dominant bacteria in the microbiome of high quality silage; they have been shown to reduce silage pH and prevent the dominance of undesirable microorganisms by producing lactic acid (Guan et al., 2018). The relatively low levels of WSC identified in the silage produced by *P. sinese* varieties in this study may have restricted the growth of LAB, resulting in the production of low quality silage.

However, another important reason for the low silage quality is the higher moisture content of *P. sinese* (77.5∼88.7%). In normal conditions, the ideal moisture content of silage materials is 65–70%, the higher moisture content is conducive to the growth of undesirable microorganisms, which decreased the silage quality. In the tropics of southern China, when wilting grass, there are often showers. In order to avoid rain damage during the silage making, in the present study, we try to prepare *P. sinese* silage with out wilted for investigating their silage quality, microbial community and the correlation with the environmental factor. One caveat with this study, the lower silage quality reminded us ensiled directly was not the suitable way for *P. sinese* preparation.

### Bacterial Community of *P. sinese* Silage

In the present study, *Proteobacteria, Firmicutes, Bacteroidetes*, and Actinobacteria were dominant in all *P. sinese* samples. These findings are consistent with previous reports examining *P. sinese*, corn stover and red clover silage (Xu et al., 2017; Dong et al., 2019; Wu et al., 2020) and have been corroborated by previous studies that reported *Firmicutes* and *Proteobacteria* as the most abundant phyla in silages, with their abundance increasing to more than 90% after fermentation (Li L. et al., 2017; Liu et al., 2019; Wang et al., 2019b). Normally, *Lactobacillus* is the major bacterial and dominant genus in well preserved silage, with *Lactobacillus* performing desirable functions during fermentation (Li L. et al., 2017; Liu et al., 2019; Wang et al., 2019b). However, in this study, *Pseudomonas*, *Massilia*, and *Raoultella* were the predominant genera, which is consistent with previous research reporting that these bacteria were associated with the unsatisfactory fermentation of silage (Li L. et al., 2017; Li et al., 2019a; Wu et al., 2020). *Pseudomonas* is considered especially undesirable due to the production of biogenic amines that reduce the protein content and nutritional value of silage (Silla Santos, 1996; Dunière et al., 2013). The present study shows that *Pseudomonas* is one of the most abundant genera of bacteria present in the silages produced by differing *P. sinese* varieties. It is proposed that *Pseudomonas* contributed to the poor *P. sinese* silage quality, as has been previously reported in studies examining forage silage (Xu et al., 2017; Dong et al., 2019; Wang et al., 2019a). However, Ogunade et al. (2018) found that *Pseudomonas* were negatively correlated with pH, ammonium nitrogen, yeast, and mold, suggesting that it may be beneficial to silage fermentation. Therefore, the underlying mechanisms of *Pseudomonas* in silage fermentation processing need to be further investigated.

### Correlations Between Bacterial Communities and the Chemical Composition

This study found that there was a significant correlation between the chemical composition of silage raw materials and silage microorganisms. The chemical composition of different varieties of *P. sinese* was quite different, so the characteristics of silage microbial community were also different. This may be explained by the fact that these microorganisms are chemoorganotrophic bacteria that produce energy through the oxidation of organic matter such as starch and organic acids. *Pseudomonas*, *Bacteroides*, and *Stenotrophomonas* all consume protein, whilst *Megamonas*, *Raoultella*, *Citrobacter*, and *Enterococcus* ferment carbohydrates (Skerman et al., 1980; Schleifer and Kilpper-Bälz, 1984; Drancourt et al., 2001; Tian et al., 2019) and *Bacteroides* and *Stenotrophomonas* can utilize either (Palleroni and Bradbury, 1993; Silla Santos, 1996; Xu et al., 2003; Dunière et al., 2013; Li M. et al., 2017). Guan et al. (2018) reported that the WSC content of raw corn correlated with the abundance of *Lactobacilli* and *Acetobacter* in the resultant corn silage. Corroboratively, Yang et al. (2019) found the same correlation, however, there was no significant correlation between DM and microorganism abundance in alfalfa silage. The present study showed significant correlation between the species of bacteria and the chemical composition of the raw *P. sinese* used to produce the silage. The results of this study further compound the significant relationship between the chemical composition of the raw material and the resultant silage. Discrepancies between this study and those mentioned above are most likely due to the differences in the chemical composition of the 12 varieties of *P. sinese* and the forage material they used.

### Correlations Between Bacterial Communities and Fermentation Characteristics

Silage microorganisms and metabolites are the key factors affecting silage fermentation quality. *Megamonas*, *Bacteroides*, and *Citrobacter* all have the ability to ferment multiple carbohydrates and produce an array of organic acids, including lactic, acetic, and propionic acid (Skerman et al., 1980; Xu et al., 2003; Li M. et al., 2017; Tian et al., 2019). Therefore, it is unsurprising that there was a significant positive correlation between the organic acid content and the microbiome of the silage. *Pseudomonas* is an undesirable bacteria in silage due to its production of biogenic amines leading to a reduced protein content (Silla Santos, 1996; Dunière et al., 2013). Furthermore, higher levels of ammonium nitrate were associated with increased abundance of *Pseudomonas*. Silage fermentation is a complex biological process which involves a large variety of microorganisms; as such, the process produces many different metabolites which can determine the fermentation quality. Variety in the microbiome of the silage alters the metabolites produced during ensiling and may contribute to the fermentation quality. In the present study, the silage produced from the different varieties of *P. sinese* lacked an abundance of *Lactobacillus*; this may explain why the fermentation quality of the silage was poor. Other studies have corroborated these findings, by showing that fermentation characteristics are highly correlated with the microbiome of the silage, influencing the overall fermentation quality (Guan et al., 2018; Ogunade et al., 2018; Ren et al., 2019; Yang et al., 2019). Therefore, reducing the content of non-lactic acid bacteria is an effective measure to improve the fermentation quality of silage.

### Correlations Between Bacterial Communities and Environmental Factors

The results of this study reveal that the microbiome of silage is affected by internal factors (chemical composition of the raw silage material) and external factors (temperature, precipitation levels). This has previously been discussed in other studies, with fermentation metabolites, precipitation, temperature, humidity, longitude, latitude and altitude all being reported to effect the microbiome of the silage and influence the fermentation quality (Muck, 2013; Bernardes et al., 2018; Guan et al., 2018). The results of this study were consistent with previous reports assessing the effect of rainfall and temperature on silage quality (Kim and Adesogan, 2006; Coblentz and Muck, 2012). Guo et al. (2014, 2015) highlighted the importance of sunlight on the fermentation of Napier grass and Italian Rye grass; the results of this study also show that sunlight affects the quality of *P. sinese* silage due to its influence on the silage microbiome. In addition, the storage temperatures of the silage influenced the bacterial diversity/fermentation quality, which had been previously reported in the silage of *Moringa oleifera* leaves (Wang et al., 2019a). Moreover, Guan et al. (2018) reported that environmental factors (temperature, humidity, and precipitation) affected the fermentation quality of silage mediated by changes in the microbiome. These results suggest that high temperature, rain, and high humidity in the south of China have a great negative influence on the microbiome of the silage from *P. sinese*. The reason that environmental factors affect the microbial community of silage may be that environmental differences affect the community structure of epiphytic bacteria on raw materials, which have a greater impact on the initial stage of silage fermentation. With the process of silage fermentation, the impact of environmental factors on the microbial community will be reduced, thus lightened its impact on silage quality. Interestingly, this study identified that longitude and latitude had significant effects on the bacterial diversity of the silage, leading to differences in the fermentation quality of the silage. This suggests that for silage preparation in different regions, geographical factors and their effect on silage microbiome must be considered in order to make provisions to ensure high quality production.

## Conclusion

The study presented herein examined the microbiome and fermentation quality of silages from the natural fermentation of 12 varieties of *P. sinese* grown across southern China. The silage quality of *P. sinese* was generally unsatisfactory with low V-Scores. There was a predominance of undesirable genera including *Pseudomonas*, *Massilia*, and *Raoultella* in silage produced from *P. sinese;* this led to poor fermentation quality. The strong correlation between the chemical composition of the silage material and the fermentation characteristics and bacterial community of the resultant silage. In addition, precipitation, temperature, humidity, and location significantly influenced the bacterial community of the silage. The specific *P. sinese* cultivar used for silage production and the environmental factors it has been subject to must be considered in order to ensure a high-quality end product.

## Data Availability Statement

The datasets presented in this study can be found in online repositories. The names of the repository/repositories and accession number(s) can be found below: https://catalog.data.gov/dataset/sequence-read-archive-sra, PRJNA624770.

## Author Contributions

ML, XZ, and DY did the experimental design work. ML, XZ, DY, and JT conducted the experiments. ML, XZ, DY, JT, HZ, and YC analyzed the data. ML and XZ wrote the manuscript. All authors read and approved the manuscript.

## Conflict of Interest

The authors declare that the research was conducted in the absence of any commercial or financial relationships that could be construed as a potential conflict of interest.

## Footnotes

1 https://docs.qiime2.org/2019.1/

2 http://www.r-project.org/

3 www.biocloud.net

## Supplementary Material

Supplementary Table 1 Environmental factors effecting the 12 varieties of *P. sinese.* MIN, *Pennisetum purpureum* Schum. cv. Gui Min Yin; TW, *Pennisetum purpureum* Schumab ev. Taiwan; MOTT, *Pennisetum purpureum* cv. Mott; KG, *Pennisetum purpureum* × *P*. *glaucum* cv. Reyan No. 4; DY, *Pennisetum purpureum* K.; HONG, *Pennisetum purpureum* Schumab cv.; JING, *Pennisetum purpureum* K. Jingmu No. 1; AX, *Pennisetum purpureum* K.; ZJ, *Pennisetum americanum* × *Pennisetum purpureum*; MZC, *Pennisetum glaucum*; PURP, *Pennisetum purpureum* cv. Purple; XC, *Pennisetum purpureum* Schumach.
