# Supplementary material for: Natural Fermentation Quality and Bacterial Community of 12 Pennisetum sinese Varieties in Southern China
Source: Front Microbiol. 2021 Apr 29;12:627820. doi: 10.3389/fmicb.2021.627820 (PMC8116707; doi:10.3389/fmicb.2021.627820)
Supplement: Supplementary Table 1 — Environmental factors effecting the 12 varieties of P. sinese. MIN, Pennisetum purpureum Schum. cv. Gui Min Yin; TW, Pennisetum purpureum Schumab ev. Taiwan; MOTT, Pennisetum purpureum cv. Mott; KG, Pennisetum purpureum × P. glaucum cv. Reyan No. 4; DY, Pennisetum purpureum K.; HONG, Pennisetum purpureum Schumab cv.; JING, Pennisetum purpureum K. Jingmu No. 1; AX, Pennisetum purpureum K.; ZJ, Pennisetum americanum × Pennisetum purpureum; MZC, Pennisetum glaucum; PURP, Pennisetum purpureum cv. Purple; XC, Pennisetum purpureum Schumach. [file Table_1.DOCX]

**Supplement Table 1** Environmental factors effecting the 12 varieties of *P. sinese.*

| City | Variety | Longitude | Latitude | Altitude  (m) | Temperature (°C) | Humidity (%) | Precipitation (mm) |
| --- | --- | --- | --- | --- | --- | --- | --- |
| Fuzhou | MIN, TW | 119°.27'E | 26°.04'N | 17 | 22 | 77 | 1359 |
| Danzhou | MOTT, KG, DY | 109°.58'E | 19°.52'N | 130 | 26 | 85 | 1816 |
| Nanning | HONG, JING, AX | 108°.27'E | 22°.78'N | 102 | 21.6 | 79 | 1300 |
| Nanjing | ZJ, MZC | 118°.89'E | 31°.32'N | 13 | 15.4 | 76 | 1106 |
| Kunming | PURP | 102°.82'E | 24°.88'N | 1900 | 15 | 74 | 1035 |
| Guangzhou | XC | 113°.27'E | 23°.15'N | 19 | 21.5 | 78 | 1700 |
